# Acupuncture in the Treatment of Upper-Limb Lymphedema: Results of a Pilot Study

**DOI:** 10.1002/cncr.28093

**Published:** 2013-04-10

**Authors:** Barrie R Cassileth, Kimberly J Van Zee, K Simon Yeung, Marci I Coleton, Sara Cohen, Yi H Chan, Andrew J Vickers, Daniel D Sjoberg, Clifford A Hudis

**Affiliations:** 1 Integrative Medicine Service, Memorial Sloan-Kettering Cancer CenterNew York, New York; 2 Department of Surgery, Memorial Sloan-Kettering Cancer CenterNew York, New York; 3 Breast Cancer Medicine Service, Memorial Sloan-Kettering Cancer CenterNew York, New York; 4 Integrative Medicine Service and Department of Epidemiology and Biostatistics, Memorial Sloan-Kettering Cancer CenterNew York, New York

**Keywords:** axillary lymphadenectomy, acupuncture, lymphedema intervention, breast carcinoma, manual lymph drainage, complex decongestive therapy

## Abstract

**BACKGROUND:**

Current treatments for lymphedema after breast cancer treatment are expensive and require ongoing intervention. Clinical experience and our preliminary published results suggest that acupuncture is safe and potentially useful. This study evaluates the safety and potential efficacy of acupuncture on upper-limb circumference in women with lymphedema.

**METHODS:**

Women with a clinical diagnosis of breast cancer−related lymphedema (BCRL) for 0.5-5 years and with affected arm circumference ≥2 cm larger than unaffected arm received acupuncture treatment twice weekly for 4 weeks. Affected and unaffected arm circumferences were measured before and after each acupuncture treatment. Response, defined as ≥30% reduction in circumference difference between affected/unaffected arms, was assessed. Monthly follow-up calls for 6 months thereafter were made to document any complications and self-reported lymphedema status.

**RESULTS:**

Among 37 enrolled patients, 33 were evaluated; 4 discontinued due to time constraints. Mean reduction in arm circumference difference was 0.90 cm (95% CI, 0.72-1.07; *P* < .0005). Eleven patients (33%) exhibited a reduction of ≥30% after acupuncture treatment. Seventy-six percent of patients received all treatments; 21% missed 1 treatment, and another patient missed 2 treatments. During the treatment period, 14 of the 33 patients reported minor complaints, including mild local bruising or pain/tingling. There were no serious adverse events and no infections or severe exacerbations after 255 treatment sessions and 6 months of follow-up interviews.

**CONCLUSIONS:**

Acupuncture for BCRL appears safe and may reduce arm circumference. Although these results await confirmation in a randomized trial, acupuncture can be considered for women with no other options for sustained arm circumference reduction. ***Cancer* 2013;119:2455-2461**. © *2013 American Cancer Society*.

## INTRODUCTION

Lymphedema, the chronic swelling of a limb that can develop after lymph node removal, is a dreaded complication of breast cancer treatment. Lymphedema is more common than secondary tumors or heart damage resulting from chemotherapy or radiation.^1^ Arm lymphedema affects approximately 30% of breast cancer survivors,^2^ and median incidence rates increase with longer follow-up, with cases presenting well beyond the active treatment period.^3^–^5^ Even minor lymphatic injury can result in lymphedema, thereby exposing many breast cancer survivors to the risk of this complication.^6^

Despite the use of less invasive surgical techniques for cancer staging, there remains a clinically relevant risk for the development of lymphedema.^6^ Further, use of radiation therapy, lymph node status, tumor burden, postoperative seroma or infection, rising rates of obesity, and an aging population each independently increases the risk of this complication.^5^,^7^

Lymphedema can have devastating consequences. Many patients have frequent arm infections, often requiring hospitalization for antibiotics. Lymphedematous arms are heavy, swollen, and stiff, with thickened, rough skin. Courses of regular, intensive physical therapy are often required to reduce the volume of lymphatic fluid in the affected arm.^8^,^9^ These treatments and subsequent maintenance regimens must be continued to prevent fluid reaccumulation or repeated when lymphedema recurs. In addition, patients are obligated to wear tight, uncomfortable elastic stockings on their arm in efforts to prevent worsening of the swelling. These garments serve as a constant reminder to patients of their cancer diagnosis, decreasing quality of life and increasing anxiety.^10^–^16^

Lymphedema is a significant source of biomedical expenditure. Managing lymphedema-related problems increases overall treatment costs by more than $10,000 a year per patient.^17^ These costs are a significant source of hardship for many patients, as insurance coverage is limited and often inadequate for the lifetime ongoing treatments required.

Early clinical trials as well as our own preliminary data have shown that acupuncture can decrease limb swelling and improve the symptoms of lymphedema in both lower and upper extremities.^18^–^21^ The value and safety of acupuncture are documented in the growing body of literature on acupuncture treatment for chronic pain,^22^–^26^ osteoarthritis,^27^ migraine,^28^ and the relief of procedural anxiety.^29^,^30^ In addition, acupuncture has been used to treat a range of problems associated with cancer and cancer treatments, such as hot flashes,^31^,^32^ chronic fatigue,^33^ neuropathy,^34^ nausea and vomiting,^35^,^36^ xerostomia,^37^ and dysphagia.^38^ Because current treatment options for lymphedema are time-consuming, expensive, and temporary, there is a pressing need for effective means to treat this problem. Therefore, this study aimed to evaluate the safety and potential efficacy of acupuncture to treat chronic upper-limb lymphedema.

## MATERIALS AND METHODS

Following institutional review board approval, we conducted a preliminary trial (http://ClinicalTrials.gov identifier: NCT01003951) to assess the safety and efficacy of acupuncture in patients with breast cancer-related lymphedema (BCRL). Patients were identified and screened and enrolled in the study following informed consent and their oncologists' approval. Inclusion criteria were: women aged ≥18 years with unilateral lymphedema, defined as ≥2 cm in circumference difference between affected and unaffected arms resulting from surgery and/or radiation therapy for breast cancer,^6^ and a clinical diagnosis of lymphedema for at least 6 months and no more than 5 years. The 6-month postsurgery time frame allowed for any surgically related nonlymphedema swelling to subside, and a cap of 5 years, which we believed to capture the broadest range of cases, has been used as a time in previous studies.^6^,^39^

Women with previous acupuncture treatment for lymphedema or currently using diuretics were ineligible. Additional exclusion criteria amended to the original protocol included metastatic cancer, history of autoimmune or fibroproliferative disorders, history of primary lymphedema or bone marrow transplant, or current treatment with corticosteroids or myelosuppressive or stimulatory drugs. Patients receiving maintenance treatment for lymphedema such as exercise, massage, or compression garments were included, as these interventions are standard practice at Memorial Sloan-Kettering and because chronic lymphedema typically persists or recurs despite such interventions.

Each patient received acupuncture treatment for lymphedema twice weekly for 30 minutes over 4 consecutive weeks (see Acupuncture Treatment Technique section). No further acupuncture treatments were given or measurements taken after the 4-week treatment period, but monthly follow-up calls were conducted for 6 months following completion of treatment to document any side effects or complications and to determine patients' self-reported lymphedema status.

Our trained research assistants asked participants the following questions, which were aimed to identify any adverse events potentially from acupuncture: 1) Have you experienced any reactions or adverse events since the last time we talked? 2) Are you currently receiving treatment for your lymphedema: exercise, arm wrapping, manual lymph drainage, pneumatic compression, use of compression garments, other? 3) How is your lymphedema since the last time we talked? Same? Better? Worse?

### Study Design

A Simon's 2-stage minimax design was used to discriminate between a 5% response rate and a response rate of 20%, which would merit further study. The design yields an 80% probability of a positive result if the response rate is ≥20%, and a 95% probability of a negative result if the true response rate is ≤5%. Acupuncture was considered promising if at least 3 of 27 study participants achieved a reduction of ≥30% in comparative arm circumference. This goal was determined on the basis of our clinical experience in the absence of any consistent definition found in the clinical literature.

Our pilot study met its goal after 4 responses occurred in 9 treated patients, and the data were published.^21^ We continued the trial to evaluate a total of 33 patients for whom data are reported here.

### Acupuncture Treatment Technique

**Figure 1:**
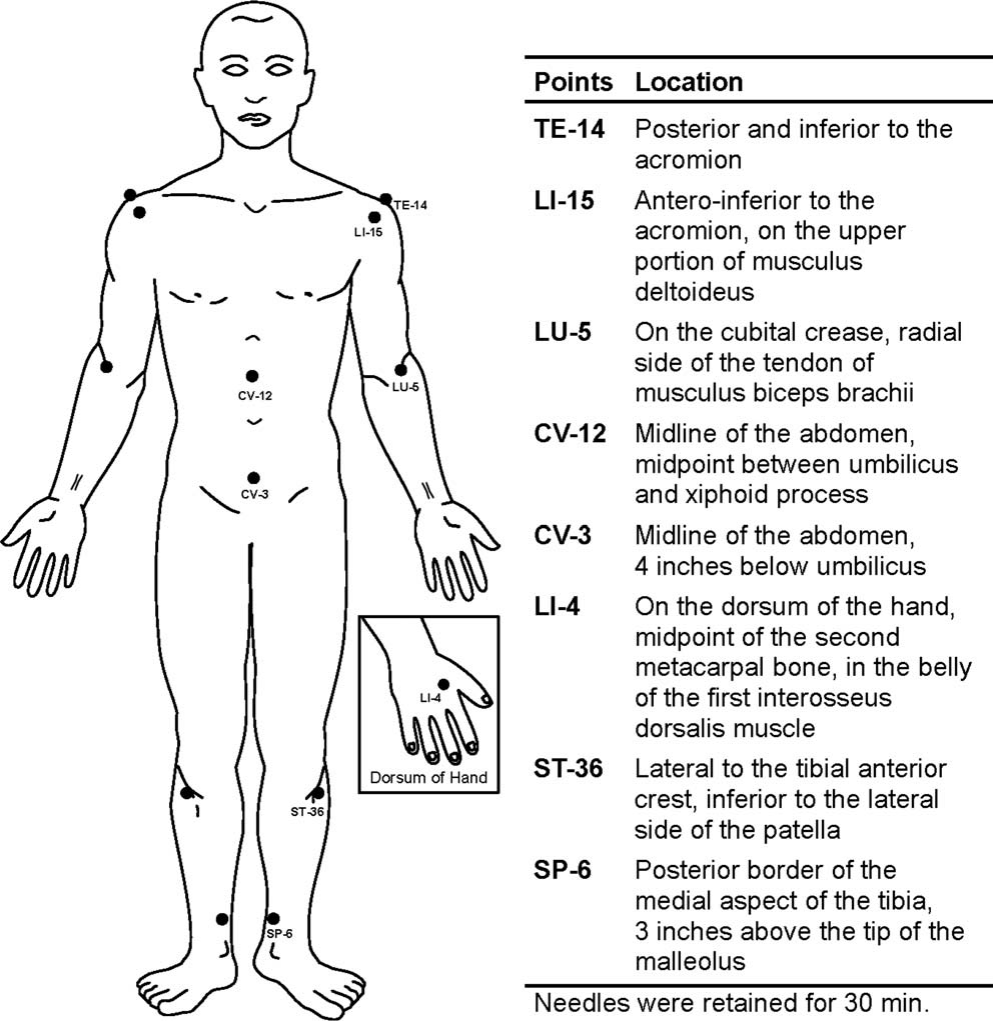
Acupuncture points used to treat lymphedema in 30-minute sessions twice weekly for 4 consecutive weeks.

Traditional Chinese medicine (TCM) acupuncture treatment was performed by licensed Memorial Sloan-Kettering Cancer Center (MSKCC) Integrative Medicine Service staff acupuncturists with at least 5 years' clinical experience in treating patients with cancer. Alcohol swabs were applied prior to insertion of sterile single-use filiform needles (32-36 gauge; 30-40 mm in length, Tai Chi brand, made in China and distributed by Lhasa OMS, Weymouth, MA) that penetrate 5-10 mm into the skin. A total of 14 needles were inserted: 4 in both affected and unaffected limbs, 2 in acupuncture points on both legs, and 2 in unilateral points on the torso. Selected acupuncture points (Fig. 1) were stimulated manually by gentle rotation of the needles with lift and thrust. The acupuncturists did not intentionally seek to achieve a *de qi* sensation.

Specific acupuncture points used in this study were determined on the basis of historical context, the published literature, and the consensus of our experienced group of MSKCC staff acupuncturists.^18^–^20^,^34^,^37^ Many of these acupoints are used to treat pain, weakness, and motor impairment; others are traditionally used to drain “dampness,” a TCM concept similar to edema.

### Lymphedema Measurement

Two-point circumference measurements of the affected and unaffected arms were performed before and after each treatment session using our previously published methods.^6^,^21^ This is the most common approach used to diagnose upper-extremity lymphedema, and sequential measurements over time that include pretreatment measures are considered to be optimal clinically.^40^,^41^ Our ipsilateral and contralateral measurements at both baseline and weekly during the course of treatment also controlled for the inherent biases that occur with straight arm circumference measures. These include weight gain or loss and arm dominance and other predispositions.^6^ Data also indicated that 2-point measures with a 5% circumference change threshold have high sensitivity and specificity (80% and 71%, respectively), close correlation to limb volume calculations using water displacement, and compare favorably with the sensitivity and specificity of multiple-point measurements with high test-retest and interrater reliability.^42^–^44^ In addition, circumference measurements provide reliable calculations for differences >0.4 cm.^45^ Further, water displacement techniques have practical difficulties such as space issues, spillage, and the need for sterile technique for each measurement.

Measurements were performed by trained research assistants 10 cm above (upper arm) and 5 cm below (lower arm) the olecranon process using nonstretch tape measures. We used our published technique^21^ to calculate the extent of BCRL by determining the difference in circumference of the affected and unaffected arms at the site with the largest baseline difference. Percent change in lymphedema was defined as:


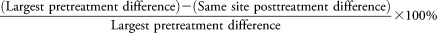


This formula enabled the determination of response to treatment and adjusted the outcomes relative to baseline arm circumference differences. The mean change in the extent of these differences was also analyzed as a continuous variable using the Student *t* test for paired data.

## RESULTS

**Figure 2:**
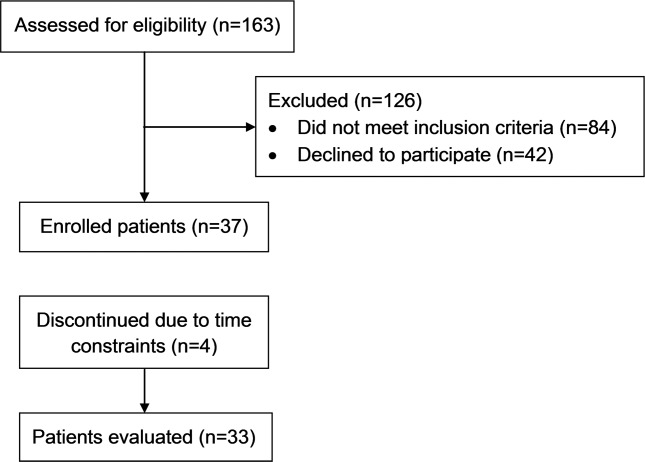
Patient disposition.

A total of 37 patients were enrolled in the study from November 2009 to May 2011 (Fig. 2). Four patients discontinued the study because of time constraints, and data for the remaining 33 patients are presented. Patient characteristics are shown in Table 1. Median age at consent was 55 years. A majority of patients had received chemotherapy and radiation therapy. There was a median of 3.9 years from patients' axillary surgery to the start of acupuncture, and most patients had been on standard lymphedema treatments before entering the study.

**TABLE 1:** Patient Demographics and Characteristics^a^

	Patients (n = 33)
Age at consent, y	55 (50-65)
Age at axillary surgery, y	51 (47-59)
Years from axillary surgery to acupuncture	3.9 (2.8-5.2)
BMI, kg/m^2^	30.4 (26.7-35.4)
Race	
White	25 (76%)
Black	6 (18%)
Asian	2 (6%)
**Previous treatments**	
Chemotherapy	29 (88%)
Radiation therapy	28 (85%)
**Primary surgery**	
Mastectomy	22 (67%)
Breast conservation	11 (33%)
**Axillary surgery**	
ALND	26 (79%)
SLNB	1 (3%)
ALND and SLNB	6 (18%)
**Affected arm**	
Left	21 (64%)
Right	12 (36%)
Standard lymphedema treatments used prior to study entry	29 (88%)

Abbreviations: ALND, axillary lymph node dissection; SLNB, sentinel lymph node biopsy.

a All values presented are median (interquartile range) and frequency (percent).

**Figure 3:**
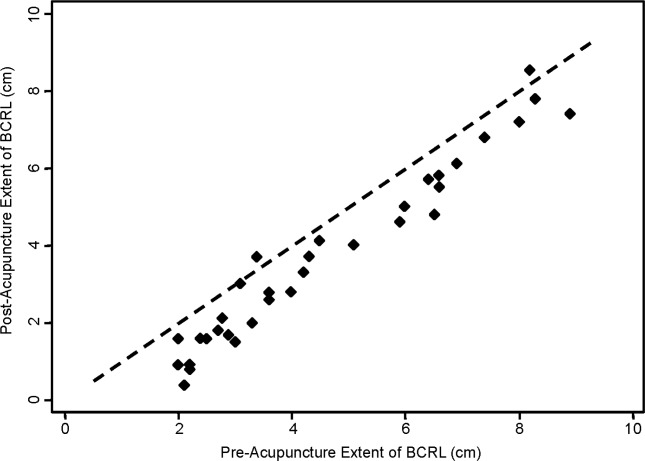
Extent of breast cancer–related lymphedema (BCRL) pre- and postacupuncture. Dashed line is 45 degrees and indicates no change in the extent of BCRL.

There were 255 acupuncture treatment sessions among the 33 evaluable patients. Twenty-five patients (76%) received all 8 sessions, 7 (21%) missed 1 treatment session, and 1 (3%) missed 2 treatment sessions. A total of 11 patients (33%; 95% CI, 18%-52%) exhibited a reduction of ≥30% in the extent of BCRL after acupuncture treatment, and more than half (n = 18; 55%; 95% CI, 36%-72%) experienced a reduction of ≥20% (Table 2). The mean reduction in the extent of BCRL was 0.90 cm (95% CI, 0.72-1.07 cm; *P* < .0005; Table 3). Reduction in arm circumference difference was observed across the full range of severity of lymphedema (Fig. 3).

**TABLE 2:** Percent Reduction in Extent of BCRL

Reduction Range	Patients (n = 33)
<0%	2 (6%)
0% to <10%	4 (12%)
10% to <20%	9 (27%)
20% to <30%	7 (21%)
30% to <40%	5 (15%)
40% to <50%	1 (3%)
50% to <60%	3 (9%)
60% to <70%	1 (3%)
70% to <80%	0
80% to 90%	1 (3%)
≥20% Reduction in extent of BCRL	18 (55%)
≥30% Reduction in extent of BCRL	11 (33%)

Abbreviations: BCRL, breast cancer-related lymphedema.

Total percentages for patients do not add up to 100% because of rounding. Extent of BCRL determined using the difference in circumference of the affected and unaffected arms at the site with the largest baseline difference.

**TABLE 3:** Mean Reduction in Centimeters (SD) in Extent of BCRL

Pretreatment	Posttreatment	Difference	95% CI	*P*^a^
4.6 (2.2)	3.7 (2.3)	0.90	0.72-1.07	< .0005

Abbreviations: BCRL, breast cancer-related lymphedema; CI, confidence interval, SD, standard deviation.

a Student *t* test for paired data; extent of BCRL determined using the difference in circumference between the affected and unaffected arms at the site with the largest baseline difference.

Four of 11 responders reported sustained improvement for 4 months during the follow-up period. Three additional responders reported sustained improvement for at least 4 weeks after treatment cessation. Only 2 patients reported using no other standard treatments for their lymphedema while in the study (1 responder, 1 nonresponder). Twenty-eight of 30 patients made no change to their standard regimens during treatment; the other 2 patients (1 with missing data) were both nonresponders. Similarly, almost all patients continued standard treatments during follow-up. One nonresponder did not have sufficient data to determine their patterns for other treatments throughout the study.

During the treatment period, 12 of the 33 patients reported mild bruising or minor pain/tingling in the arm, shoulder, or acupuncture site at least once (Table 4). One patient experienced a transient (4-day) increase in lymphedema in the axilla of the lymphedematous arm. There were no serious adverse events—no infections or severe exacerbations—after 255 treatment sessions. Similarly, there were no treatment-related infections, severe exacerbations, or other serious adverse events during 6 months of follow-up interviews.

**TABLE 4:** Nonserious Treatment-Related Adverse Events Reported During the 4-Week Treatment Period and 6-Month Follow-Up

Adverse Event^a^	Patients (n = 33)^b^
**During the 4-week treatment period**	
Bruising (absence of Gr 3/4 thrombocytopenia)^c^	9 (27%)
Edema: limb	1 (3%)
Nausea	1 (3%)
Neuropathy: sensory	1 (3%)
Pain: extremity-limb	2 (6%)
**At 4 weeks through 6-month follow-up**	
Bruising (absence of Gr 3/4 thrombocytopenia)	0 (0%)
Edema: limb	0 (0%)
Nausea	0 (0%)
Neuropathy: sensory	0 (0%)
Pain: extremity-limb	1 (3%)

a All adverse events were determined to be possibly or unlikely to be related to treatment except for bruising, which was deemed to be definitely treatment related. There were no probable treatment-related adverse events.

b Patients who reported the same type of adverse event more than once were tabulated only once.

c Minor bruising occurred at the acupuncture site.

## DISCUSSION

This study demonstrates that acupuncture for the treatment of BCRL is safe and well tolerated. We saw evidence that acupuncture decreases lymphedema, as shown by the reduction of ≥30% in one-third of patients and the significant mean reduction in the extent of these differences. Whether acupuncture alone was responsible for this reduction was not evaluable in this pilot study. Our focus was on safety and potential efficacy, as current clinical practice to protect the lymphedematous arm prohibits needling. Yet data on the use of acupuncture in lymphedematous arms from Japan and from the United States found neither infections nor other side effects.^18^,^46^,^47^

Moreover, 2 large prospective studies from Germany indicated that acupuncture for various indications was safe when performed by qualified practitioners.^48^,^49^ Among 229, 230 patients and more than 2.2 million acupuncture sessions in the study by Witt et al,^48^ the adverse events reported in 8.6% of sampled patients were mostly minor and included bleeding and bruising. Melchart et al^49^ found only 6 cases of serious adverse events after 760,000 acupuncture treatments in 97,733 patients.

In our study, we observed no infections, severe exacerbations, or other serious adverse events throughout the treatment period and for 6 months thereafter. The high treatment adherence rate among study participants is also encouraging. The varying degrees and tapering of self-reported sustained improvement during the 6-month follow-up period pose several questions. Is improvement due to acupuncture alone? Is it more effective in some patients than others? Might a maintenance regimen component lengthen the duration of response? Our recently opened randomized trial will answer some of these questions. However, the finding that most patients continued their use of standard treatments during the follow-up period and the corresponding tapering of improvement during that period suggest that acupuncture was responsible for the benefits experienced.

Existing interventions such as complex decongestive therapy are expensive and require significant resources.^8^,^17^,^50^ Acupuncture treatment appears to work safely and effectively in the hands of acupuncturists who are properly trained to treat patients with cancer. There is some risk of infection with lymphedematous tissue in general. However, there are no documented cases of lymphedema infection from acupuncture. This is likely because cancer patients receiving acupuncture in lymphedematous limbs participate in clinical trials involving treatment by qualified practitioners under sterile conditions. Such prerequisites are critical to the use of acupuncture in cancer patients for the treatment of lymphedema or any other symptom.

Should a patient on lymphedema treatment develop sudden onset of infection, all modalities, including manual lymphatic drainage, bandaging, pumps, wearing compression garments, and acupuncture, should be discontinued until the infection is cleared. The treating physician should be contacted as soon as possible.

The therapeutic and cost-reduction potential of acupuncture for lymphedema may yield an important tool in the arsenal of lymphedema management. Although randomized clinical trial results await, including our ongoing study, acupuncture can be considered to treat this distressing problem confronted by many women with no other options for sustained reduction in arm circumference.
